# A Survey on Deep Learning Techniques for Fingerprint Presentation Attack Detection

**DOI:** 10.3390/s26041283

**Published:** 2026-02-16

**Authors:** Hailin Li, Raghavendra Ramachandra

**Affiliations:** SAFE Center, Norwegian University of Science and Technology (NTNU), 2815 Gjøvik, Norway

**Keywords:** biometrics, deep learning, fingerprint presentation attack detection

## Abstract

The vulnerabilities of the fingerprint authentication system have raised security concerns in terms of adapting them in highly secured access control applications. Therefore, fingerprint presentation attack detection (FPAD) methods are essential to ensure reliable fingerprint authentication. Due to the lack of generalization of the traditional handcrafted-based approaches, deep learning-based FPAD has become mainstream and achieves remarkable performance in the past decade. In this paper, we will concentrate only on deep learning-based FPAD methods. We investigate recent methods and divide those into different categories to provide a comprehensive description. The benchmark metrics and publicly available datasets are also discussed. Lastly, we conclude the paper by discussing future perspectives to inspire further research.

## 1. Introduction

Biometric verification is widely deployed in numerous access control applications including border control, forensic science, smartphone access, and attendance systems. Biometric systems can be designed using either physiological traits (e.g., fingerprint, face, iris), behavioral traits (e.g., gait, keystroke, voice) or a combination of both. Among various physiological traits, the face, iris, and fingerprint have dominated the majority of applications because of their reliability and accuracy in performance, which can be attributed to the uniqueness of these biometric characteristics. However, fingerprint biometric is one of the traditional biometric characteristics in various applications, considering the reliability of finger patterns over a long period of time and the representation and matching of the features, which can achieve billions of fingerprint comparisons in a single second with high accuracy [1].

**Figure 1:**
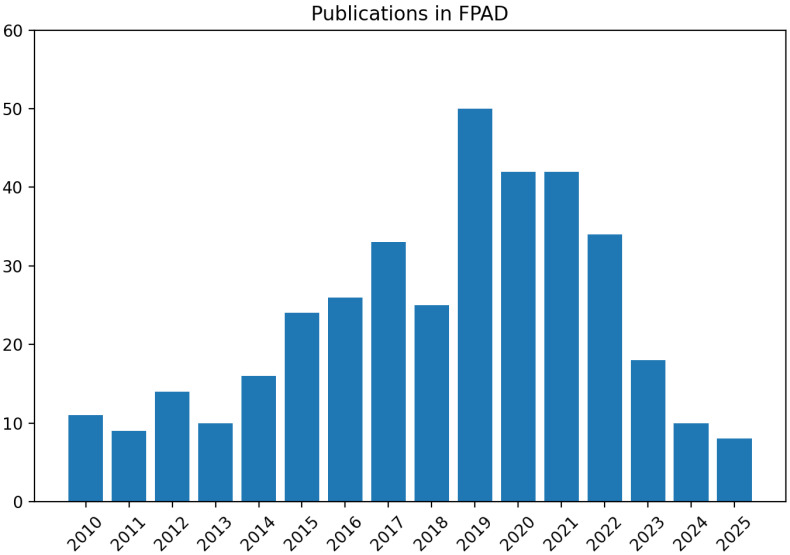
Publications of FPAD in recent years obtained through Google Scholar search with keywords: “fingerprint spoof detection”, “fingerprint presentation attack detection”, and “fingerprint liveness detection”.

Figure 1 summarizes the growth of publications on fingerprint presentation attack detection (FPAD) over recent years, motivating the need for a structured and security-centric survey.

Fingerprint images are captured using contact and contactless sensing, among which contact-based sensing is predominantly used. Contact-based fingerprint sensing includes optical, capacitive, and ultrasonic sensors that are available as stand-alone and/or integrated devices in smartphones. Optical fingerprint scanners are the oldest type of fingerprint pattern capture. These sensors typically have a very high number of diodes per inch to capture the fine details of the finger, and the optical camera has a finite resolution. Capacitive sensors use arrays of small capacitor circuits to capture fingerprint data. Since capacitors store electrical charges, connecting them to conductive plates on the scanner’s surface allows them to track the details of a fingerprint. Ultrasonic sensors have recently been introduced to scan fingerprints, particularly in smartphones. These sensors employ an ultrasonic transmitter that can send the pulse against the finger and record the echoes used to construct the fingerprint. Further, scanning for extended periods allows additional depth data to be captured, resulting in a 3D fingerprint. There are two types of contactless sensing: (a) custom industrial cameras and (b) off-the-shelf smartphone cameras used to capture fingerphotos. The use of custom cameras for contactless capture allows designers to include multispectral cameras and other sophisticated cameras to ensure reliable verification with resilience to presentation attacks. However, fingerprint capture using contactless sensing introduces additional challenges such as uncontrolled poses, low-quality fingerprints, environmental noise, and degraded performance.

The widespread deployment of the Fingerprint Recognition System (FRS) has raised concerns about the system being attacked and attackers maliciously gaining access to the fingerprint system. The FRS can be attacked in two ways (a) direct attack and (b) indirect attacks. Direct attacks typically target the sensors of the FRS using a Presentation Attack Instrument (PAI), which is presented to the sensor to gain access. An indirect attack aims to attack the biometric subsystem components to modify the functionality. Compared with direct attacks, indirect attacks require special skills and knowledge of the biometric system to successfully gain access to the FRS. Therefore, direct attacks on the FRS have been used extensively in real-life scenarios. One real-life example of hacking the FRS using direct attacks, mainly on the national population registry, was reported in [2]. The Aadhaar-enabled Payment System (AePS) was spoofed to withdraw money from various victims. Attackers achieved this by collecting the fingerprints of the victims from registry papers that have ink-prints of fingerprints. The attackers then created a polymer fingerprint attack instrument that was used to withdraw money through AePS. Therefore, the detection of presentation attacks is of paramount importance in ensuring the security of the FRS to facilitate reliable verification.

To make the scope and positioning of this survey transparent, Table 1 reports the number of articles retrieved per database, and Table 2 contrasts this manuscript with closely related prior FPAD surveys.

### Systematic Review Protocol

To improve methodological rigor and reproducibility, a PRISMA-inspired review protocol was followed. The search was conducted in ScienceDirect, Scopus, IEEE Xplore, and arXiv using combinations of the following keywords: “fingerprint” AND (“presentation attack” OR “spoof” OR “liveness”) AND (“deep learning”). Records were screened in three stages: (1) title/abstract screening to remove non-FPAD, non-fingerprint, or non-technical articles; (2) full-text eligibility screening to retain papers that propose or evaluate FPAD algorithms; and (3) quality checks to exclude papers with missing experimental details, duplicated results, or non-reproducible reporting. For each included study, the following variables were extracted to enable cross-paper synthesis: sensing modality (contact/contactless/smartphone), attack instrument, backbone, dataset(s), evaluation protocol, metrics, and computational aspects.

Table 2 is discussed below to clarify how the present survey differs from and extends prior FPAD reviews.

Presentation Attack Detection (PAD), a.k.a anti-spoofing methods, has been widely investigated for fingerprint biometrics, resulting in several PAD techniques. The progress in Fingerprint PAD (FPAD) techniques is shown in Figure 1, which illustrates the increased interest of researchers in developing FPAD techniques. Early works on developing FPAD techniques were based on handcrafted features, in which texture-based features (Local Binary Patterns (LBPs), Binarized Statistical Image Features (BSIFs), etc.) have been widely employed in designing FPAD. However, owing to the limitations of these texture features to generalize across different types of PAIs, researchers have started to investigate FPAD using deep learning. Furthermore, the importance of FPAD has also resulted in the development of the competition platform LivDet [12], which allows participants to submit FPAD algorithms for independent evaluation.

**Figure 2:**
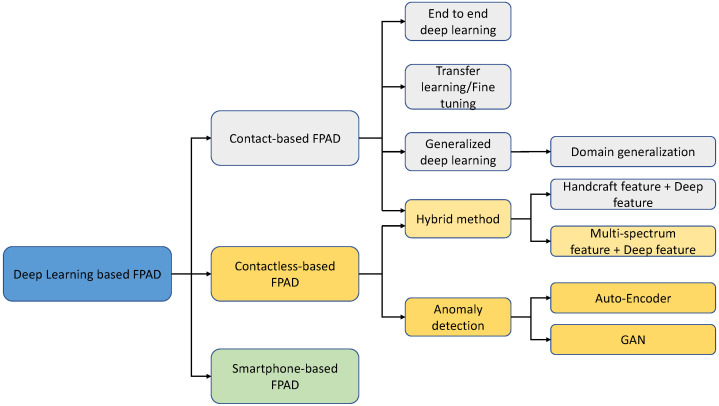
Taxonomy of deep learning-based fingerprint presentation attack detection.

The exponential growth of FPAD algorithms over the years has resulted in several survey papers, as listed in Table 2. However, multiple recent surveys already cover deep-learning-based FPAD (e.g., CNN-based detectors, GAN-based synthesis/defense, and domain generalization), and this manuscript does not claim that deep learning has been ignored in prior work. Instead, the contribution of this survey is to (i) unify contact, contactless, and smartphone sensing under a single taxonomy that is explicitly linked to deployment constraints, (ii) provide a systematic and reproducible paper-selection protocol, and (iii) synthesize cross-scenario insights on robustness, computational cost, and security threat models that are often treated separately. Hence, in this paper, we are motivated to present a comprehensive review of deep learning-based fingerprint presentation attack detection, in which we discuss recent progress, competition, performance evaluation metrics, and future work. We select the data range between 2012 and 2024 as shown in Table 1. Furthermore, we have included Figure 2, which summarizes our taxonomy of deep-learning FPAD methods across contact-based, contactless, and smartphone-based sensing. We use this taxonomy to structure the survey that follows and to map techniques to the evaluation protocols and datasets summarized later.

We also formulate several research questions as below:

- **RQ1**: How many types of FPAD methods in terms of capture device are included?
- **RQ2**: What DL techniques are used in the FPAD methods?
- **RQ3**: Which publicly available datasets are currently used in FPAD?
- **RQ4**: What are the current challenges and the future trends of FPAD techniques?

The main contributions of this study are as follows:

- We present the comprehensive survey on deep-learning-based FPAD techniques, together with the taxonomy, for both contact and contactless fingerprints, and compare these approaches on the different attributes of design;
- We present a comprehensive survey on the PAIs that are widely employed in both contact and contactless fingerprint biometrics;
- We present a study on the usage of deep learning interpretation tools on FPAD methods;
- We outline the main challenges and the potential future work for reliable fingerprint detection.

The rest of the paper is organized as follows. Section 2 presents the pipeline of a fingerprint recognition system and indicates how the FPAD system can be adjusted in the overall system. Section 3 provides a comprehensive report on the different types of PAIs for both contact and contactless fingerprints. Section 4 introduces the most common publicly available FPAD dataset. Section 5 presents a comprehensive survey of fingerprint presentation attack detection based on deep learning. Section 6 introduces the importance of deep learning model explanation and how AI interpretation methods influence the existing FPAD methods. Then, Section 7 includes the most common PAD performance evaluation metrics from the ISO standard and the LivDet competition. Section 8 discusses the open challenges and potential future research directions. Finally, we conclude this review in Section 10.

## 2. Fingerprint Recognition Systems (FRS)

**Figure 3:**
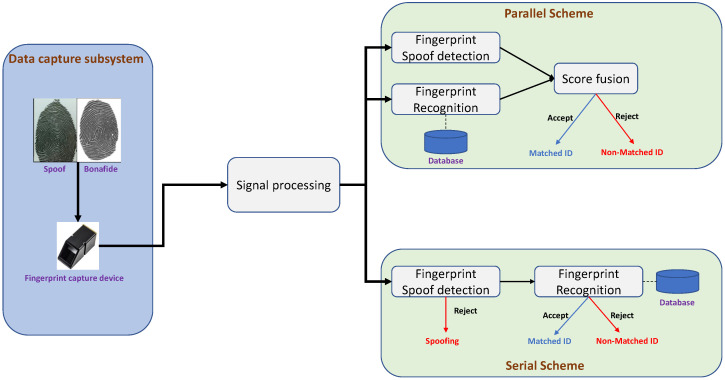
Block diagram of fingerprint verification system with PAD.

Figure 3 shows the block diagram of the fingerprint verification system. Given a fingerprint image, the signal processing unit performs various operations, including data preprocessing, quality checking, feature extraction, and template creation. Fingerprint feature extraction techniques can be level 1 (pattern), level 2 (minutiae points), and level 3 (pores and ridge shape). The common and successful fingerprint features that are widely deployed in commercial applications are based on Level 2 features. Final decisions are made using a comparator that can compare the enrolled fingerprint with the probe fingerprint image to output the comparison score. The comparison score is then compared with the preset threshold to make the final decision. The PAD system can be integrated into a fingerprint recognition system, either parallel or serial to the fingerprint comparator. In a parallel system, both the PAD and comparator perform processing independently to obtain the decisions that are combined to make the final decision. However, in a serial system, the PAD and fingerprint comparator operate sequentially. The fingerprint is first processed through the PAD unit, and if the output of the PAD unit indicates a bona fide, then the fingerprint template is passed through the comparator to make the final decision. For more detailed information on the fingerprint systems, readers can refer to [13].

## 3. Fingerprint Presentation Attack Instrument (PAI)

**Figure 4:**
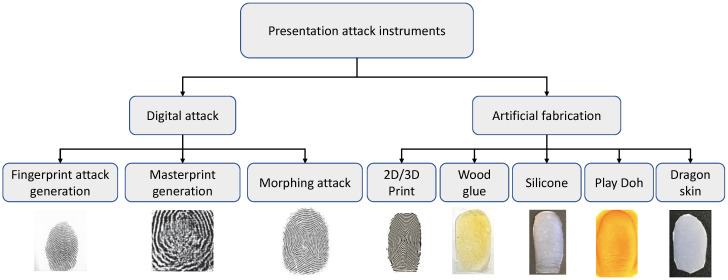
Taxonomy of fingerprint Presentation Attack Instrument (PAI).

The success of a presentation attack on fingerprint systems depends on the high-quality generation of PAI. Figure 4 shows the taxonomy of the existing PAI types commonly studied in the fingerprint literature [13]. Available PAIs can be broadly categorized into two main types: (a) digital generation and (b) artificial fabrication. Digital attack generation is performed using a computer program in which the attacks are synthetically generated using deep learning methods [14] or the custom algorithms [15,16,17,18]. Digital attacks can be used as injection attacks and/or to generate physical artifacts that can be used as presentation attacks. Examples of digital attacks include synthetic fingerprint attacks [19], master print [20] and morphing attacks [21,22]. The artificial fabrication method uses the target fingerprint impression to generate a physical artifact that can be used as a presentation attack. Artificial fabrication methods can be broadly categorized into 2D/3D printing and gummy fingers. The 2D/3D print, in which the physical artifacts are printed fingerprints, can be used as presentation attacks [23,24,25]. The gummy fingerprints are made of various materials (gelatin, Play-Doh, silicone, etc.) that may contain a specific fingerprint template and have been shown to pose potential risks to the FRS [26]. Table 3 lists the different features of the digital and artificial fabrication types of PAI generation.

**Figure 5:**
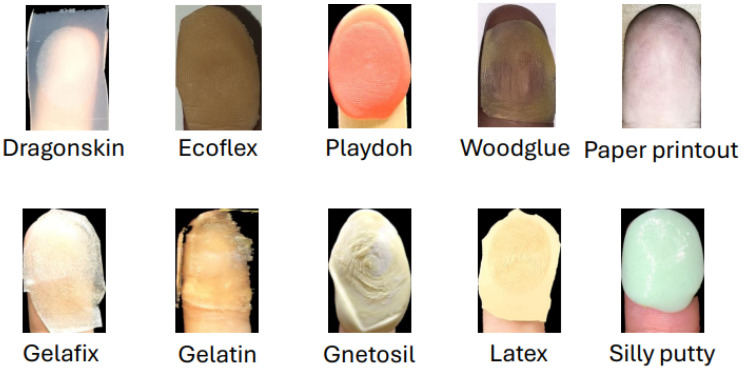
Examples of PAIs.

The common PAI examples are shown in Figure 5. In accordance with the experiments under the fingerprint verification application, the digital synthetic fingerprint shows high attack potential against the FRS.

## 4. Existing Datasets for Fingerprint PAD

A large-scale dataset is required to achieve better results when using deep-learning-based methods during both the training and testing phases. In this section, we summarize the publicly available FPAD datasets containing the data amount, subject numbers, bona fide/attack amount, and used PAI species. The most common datasets were obtained from the Fingerprint Liveness Detection Competition (LivDet) series from 2009 to 2021. Readers can refer to a review of the LivDet series [27] for detailed information on the LivDet challenge. In the first edition of the LivDet 2009 dataset, three optical scanners, Crossmatch, Identix, and Biometrika, were included with gelatin, silicone, and Play-Doh as spoof materials. In LivDet 2011, four sub-datasets were based on four different optical scanners: Biometrika, Digital Persona, ItalData, and Sagem. In addition, more PAIs, such as Silgum, Ecoflex, wood glue, and latex, are included than in LivDet 2009. The first non-optical scanner was introduced in the competition, in which the spoof materials were replenished with body doubling and modasil. In LivDet 2015, with the raised concern of FPAD methods against unseen attacks, the competition included some unknown materials for evaluation in the test dataset. Additionally, liquid Ecoflex and a two-component silicone rubber (RTV) are included in the Green Bit, Biometrika, and Digital Persona datasets, whereas the silicone rubber OOMOO is included in the Crossmatch dataset. In LivDet 2017, the competition focused on the impact of the FPAD based on user-specific effects and operator skills in fabricating replicas. In the training set, the spoofing is made of wood glue, Ecoflex, and body double, whereas gelatin, latex, and liquid Ecoflex compose the test set in order to stimulate the completely unseen scenario. Furthermore, two sets of people with different manufacturing spoofing abilities were involved in spoofing. In contrast to the previous editions, some subjects were included in the training and testing datasets to explore the impact of user-specific effects. LivDet 2019 utilized the same scanners as in the previous edition, but presented multi-material combinations, both of different consistencies and of different natures for the first time. LivDet 2021 only consists of two scanners, GreenBit and Dermalog, in which the consensual approach and the new pseudo-consensual method, ScreenSpoof, are included. Novel materials RProFast, Elmers glue, gls20, and RPro30 were chosen for the fakes. In the most recent LivDet 2023, four datasets correspond to two known sensors, Green Bit and Dermalog, and two unknown capture devices.

Besides the LivDet datasets, there are also many proposed methods that include custom datasets that are publicly available. In the Tsinghua dataset [28], the Capacitive device Veridicom is utilized as the capture device and the attack samples are created using Silicone. The samples are acquired from 15 volunteers from Tsinghua University. Two fingers are captured for each participant and ten image sequences are recorded for each real finger. Forty-seven fake fingers are manufactured with ten image sequences for each finger. The BSL dataset [29] is a collection from the Biometric System Laboratory of the University of Bologna, which contains more subjects and samples using four different PAIs. For each of 45 volunteers, the thumb and forefinger of the right hand with 10 image sequences are recorded. Instead of making whole 3D fingers, the obtained fabrications are focused on the fingertip area. The ATVS dataset utilizes silicone and Play-Doh as materials and involves 17 subjects. Two scanners are used in the Precise Biometrics dataset, which comprises 100 subjects and 500 attack samples produced using five different materials. In the Precise Biometrics Spoof-Kit (PBSKD) [30], 900 spoof fingerprint images are fabricated using 10 different types of spoof materials. Another dataset, MSU-FPAD, is also proposed in [30], with up to 9000 live samples and 10,500 spoof samples included, using these two readers and four different spoof fabrication materials. In terms of fingerphoto datasets, Taneja et al. [31] first released their fingerphoto spoofing dataset, which contains 4096 bona fide images and 8192 spoofed samples. Recently, Kolberg et al. [32] published a new dataset, COLFISPOOF, based on contactless fingerphoto, which contains 72 different PAI species. All the fingerprints for the PAIs are generated using fingerprint synthetic algorithms. The details of each dataset are listed in Table 4.

Although Table 4 aggregates widely used datasets, several limitations remain: (i) Demographic coverage is often narrow, with limited age/skin-type variability, which can bias texture statistics. In LivDet-style datasets (e.g., LivDet 2017) capture conditions introduced by different users/operators can systematically alter ridge contrast and noise, so FPAD models may exploit subject- or acquisition-specific cues rather than spoof cues. This can inflate results when subjects/sessions overlap across splits and typically becomes evident under subject-disjoint or session-disjoint evaluation. Hence, beyond overall APCER/BPCER, reporting cross-user variability helps quantify demographic/user bias rather than only stating it qualitatively. (ii) Acquisition protocols (lighting, contact pressure, moisture) vary across labs, producing domain shift that inflates in-domain and depresses cross-domain performance. (iii) Sensor heterogeneity (FTIR vs. SWIR/LSCI vs. camera phone optics) leads to markedly different signal formation; models trained on one spectrum/device frequently fail to transfer. (iv) PAI distribution is skewed toward a subset of materials; as a result, unknown-material performance (cross-material) is a persistent challenge. We therefore encourage reporting standardized metrics on public benchmarks (e.g., LivDet 2011–2021) with explicit cross-sensor and cross-material protocols.

## 5. Deep Learning-Based Fingerprint Presentation Attack Detection

In this section, we discuss the Fingerprint PAD (FPAD) algorithms presented for contact, contactless, and smartphone-based fingerprint sensing. The FPAD aims to detect whether a given fingerprint image is a bona fide or presentation attack.

### 5.1. Structured Comparison Across Scenarios and Deployment Constraints

To help practitioners navigate the diverse FPAD literature, Table 5 summarizes representative deep-learning paradigms by sensing scenario (contact, contactless, smartphone) and highlights the typical trade-offs among robustness, computation, and deployment constraints. In addition, the discussion in this section explicitly links architectural choices (e.g., shallow texture-sensitive CNNs vs. deeper backbones, and domain-generalization objectives) to the characteristics of bona fide vs. attack artifacts that differ across sensors and PAI materials.

### 5.2. Adversarial Robustness and Security Threat Models

Because FPAD is inherently security-critical, the reviewed methods are now discussed under explicit threat models: (a) *physical* adaptive attacks (improved materials, higher-fidelity molds, replay to contactless sensors) and (b) *digital* adversarial attacks (gradient-based perturbations, input transformations, or generator-assisted attacks). The survey summarizes common defenses (adversarial training, input randomization, feature denoising, ensemble defenses, and sensor-level challenge-response where available) and emphasizes that robustness must be evaluated under cross-material and cross-sensor protocols rather than only intra-dataset splits.

FPAD techniques can be broadly classified into two main categories: (a) hardware-based approaches and (b) software-based approaches. Hardware-based approaches are designed to extract liveness cues that require explicit (or dedicated) sensors to be integrated into a conventional contact fingerprint biometric system. Some of the widely used liveness measures include the capture of blood flow [45], electro-tactile [46], and pulse oximetry [47] data to detect whether the fingerprint is alive. Over the past few years, new expensive sensors, such as optical coherence tomography (OCT), have evolved. OCT is an imaging technique that allows for some of the subsurface characteristics of the skin to be imaged and extracts relevant features of multilayered tissues up to a maximum depth of 3 mm [48,49,50]. Furthermore, several contactless fingerprint capture devices such as multispectral and 3D capture devices can inherently capture the signature of liveness. The software-based approach refers to algorithms that detect whether the presented fingerprint is a bona fide or a presentation attack, irrespective of the capture device. Software-based FPAD can be further broadly divided into two types: handcraft-based and deep learning. Handcrafted-based methods refer to conventional feature representations that include techniques used to extract features such as gradients, textures, and micro-textures. The handcraft-based approach is mostly applied to contact-based fingerprint images. From fingerprint images, microtextural features can be computed using Scale-invariant feature transform (SIFT) [51], Binarized Statistical Image Features (BSIFs) [52], Local Binary Pattern (LBP) [53], and Local Phase Quantization (LPQ) [54]. Many handcraft-based approaches, such as [55,56,57,58,59], have achieved promising performance in recent years. However, handcrafted-based approaches may be limited in that the extraction process becomes difficult owing to variations in the acquired fingerprint image quality. These challenges are tackled using Deep Neural Network (DNN) terms, such as deep learning, which hierarchically learns deep-level features from images. With the rapid development of graphical processing units, the training of large-scale models has become possible. In 2012, Krizhevsky et al. [60] trained a network to classify 1.2 million high-resolution images in the ImageNet LSVRC-2010 contest into 1000 different classes that achieved huge success, which started a revolution in the computer vision field. Subsequently, training a deep convolutional neural network (CNN) has dominated image-classification tasks in various applications.

### 5.3. Contact-Based FPAD

Benefiting from the rapid development of robust CNN architectures [61,62,63] as well as advanced regularization techniques [64,65], researchers have paid more attention to employing deep neural networks to detect fingerprint attacks reliably. Many successful contact-based FPAD models rely on convolutional layers that are strongly sensitive to micro-texture and ridge-valley regularities, which are often disrupted by common PAI fabrication processes (e.g., air bubbles, material granularity, or surface reflectance artifacts). Deeper backbones can capture higher-level context but may also learn sensor- or dataset-specific shortcuts; therefore, several works report improved robustness by combining local texture cues with regularization (data augmentation, frequency-domain branches, or domain-aware normalization).

Therefore, instead of extracting texture features through handcrafted-based descriptors, deep-learning-based approaches can learn deep features that directly map fingerprint inputs to spoof detection. The traditional CNN architecture comprises convolutional layers and a pooling layer that convolves several filters to map the input images to deep-learnable features. The extracted features can be further fed into a fully connected layer for classification tasks. CNNs are well-suited for FPAD because spoof cues are often local and repetitive (e.g., pore absence, ridge discontinuities, surface sheen), and the learned convolutional filters naturally specialize to such patterns. As shown in Figure 2, deep-learning-based methods can be generally divided into two categories. Supervised learning is a straightforward way to determine bona fide and PA as a binary classification task. However, these approaches may not be generalizable to unseen domain attacks (i.e., unknown presentation attacks). Many researchers have considered generalized deep-learning models that can achieve domain generalization to enhance the generalization capacity against unseen PA types.

Initially, Nogueira et al. [66] first introduced a conventional network for fingerprint feature extraction. They trained a Support Vector Machine (SVM) classifier to detect presentation attacks based on CNN deep features and LBP features. To improve the classifier’s performance, several data augmentation techniques such as frequency filtering, contrast equalization, and Region Of Interest (ROI) filtering have been applied. Through comparison, the experiments indicate the high classification accuracy of the CNN model, which leads to a new direction that inspires more researchers to concentrate on using deep learning-based approaches on FPAD tasks. However, feature extraction and classification tasks are separated into two different parts, so the model is not optimized simultaneously.

#### 5.3.1. End-to-End Deep Learning

**Figure 6:**
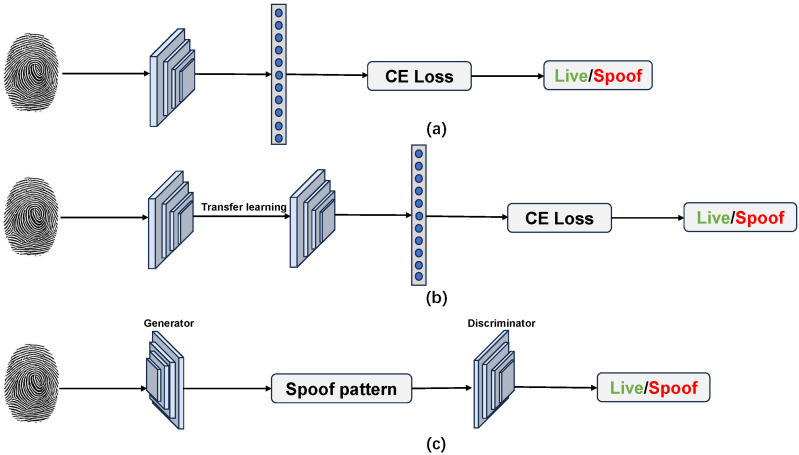
Deep learning frameworks for contact-based FPAD. (**a**) End to end deep learning model using cross-entropy loss. (**b**) Transfer learning/fine-tuning-based FPAD approach. (**c**) FPAD using generalized deep learning model.

As shown in Figure 6, end-to-end deep learning models were trained to automatically outperform classification tasks. The feature representation of the fingerprint image was extracted from the convolutional layer and passed into the fully connected layer to calculate the liveness probability using the softmax function. Table 6 presents a short description of existing end-to-end deep learning techniques.

In a study from 2015, Wang et al. [67] divide the input labeled fingerprint images into 32×32 pixel non-overlapped patches and pass them into the CNN model for training. Then, the authors adopt a voting strategy to integrate the labels of all patches to finally determine the result. Similarly, Park et al. [68] propose extending Wang’s work to reduce the processing time. Instead of extracting patches from the entire image, the authors indicate that it is more efficient to extract patches from the effective fingerprint area. Thus, after extracting patches with normal probability positions of segmented fingerprint regions, the authors train those patches with a CNN and make the decision using a voting strategy to make the classification. Menotti et al. [69] evaluate the effectiveness of the proposed newly Derived CNN architecture for spoofing detection terms using *SpoofNet*. Kim et al. [70] present an FPAD method based on a Deep Belief Network (DBN) with a series of constrained Boltzmann machines connected. DBN learns hierarchical generative features that can capture micro-texture and ridge-flow statistics from limited labels, which historically aligned with FPAD’s emphasis on material-driven textural artifacts and ROI-focused cues. The DBN is trained in two steps. Firstly, it is trained on a set of examples without supervision from the first layer to the penultimate layer to probabilistically learn the reconstruction of its inputs. Then, the model will be further trained using labeled data to perform the classification.

With an increasing number of publicly released datasets, Chugh et al. [71] utilize the Inception-v3 CNN [72] model implemented using the TF-Slim library with a replacement of the multiple-class softmax layer with a two-unit layer for the two-class problem. Then, the model is trained with local patches extracted around the fingerprint minutiae, since local patches around these minutiae are able to provide significant cues to distinguish a spoof fingerprint from live fingerprints. Along with score-level average fusion, this method evaluates several experiments such as intra-sensor and the same material, intra-sensor and cross-material, cross-sensor, and cross-dataset scenarios to consider both known and unknown attacks, which demonstrate a good average classification error (ACE). In the next work, Chugh et al. [30] further present a Fingerprint Spoof Buster based on the MobileNet-v1 [73] model trained using local patches that are centered and aligned around minutia points, and define a global *Spoofness Score* to integrate the local Spoofness Score to determine the PA.

However, FPAD methods based on CNN suffer from generalization and high computational cost problems. The selection of PA materials used in training (known PAs) directly affects the performance against unknown PAs. Some materials (e.g., EcoFlex) have been reported to be easier to detect than others (e.g., Silgum) [30]. Hence, to further investigate the better representation of different PA materials, a new dataset, namely MSU-FPAD v2.0, which combines the MSU-FPAD v1.0 and Precise Biometrics Spoof kit [30], was presented in [26]. Specifically, the database is constructed using 12 different PAIs. Then, the leave-one-out protocol is adopted; one PAI is excluded from the training set at every iteration and utilizes the 3D t-SNE technique to visualize the characteristics. Through experiments, silicone, 2D paper, Play-Doh, gelatin, latex body paint, and Monster Liquid Latex are observed to cover the entire feature space around the bona fide. Furthermore, it is considered a challenge to integrate existing deep learning-based algorithms with millions of parameters into an embedded or mobile device. Nguyen et al. [74] present the FPAD technique following the architecture of the Fire module of SqueezeNet [75] and introduce the Gram Matrix [76] to form the structure of the basis of the proposed fPADnet. This model only contains 0.3 million parameters, which is 2.4 times smaller than that of the original SqueezeNet. Similarly, Park et al. [77] introduced a fully convolutional neural network that uses the fire module of SqueezeNet as the foundation. The model can interfere with images of any size since it has no fully connected layer. The model takes the patch extracted from the input image as input and outputs three values that show the probabilities of the classes (live, false, and background) to which the patch belongs. Additional experiments with different input patch sizes at 32×32, 48×48, where 32×32 and 64×64 are evaluated to identify the optimum patch size to achieve the highest detection accuracy.

**Table 6:** Existing contact-based FPAD using end to end deep learning.

Author	Year	Backbone	Loss Function	Main Contribution
Nogueira et al. [66]	2014	DCNN	SVM	Deep feature with SVM
Wang et al. [67]	2015	DCNN	Binary CE loss	Voting strategy
Menotti et al. [69]	2015	DCNN	Binary CE loss	*SpoofNet*
Kim et al. [70]	2016	DBN	MSE loss	Deep Belief Network
Park et al. [68]	2016	DCNN	Binary CE loss	Patch-based method
Lazimul and Binoy [78]	2017	DCNN	Binary CE loss	Fingerprint Image Enhancement
Jang et al. [79]	2017	DCNN	Binary CE loss	Contrast enhancement and CNN
Chugh et al. [71]	2017	Inception-v3	Binary CE loss	Extract patch near minutiae
Chugh et al. [30]	2018	MobileNet-v1	Binary CE loss	Define a global *Spoofness Score*
Pala [80]	2017	DCNN	Triples loss	Triplet embedding representation
Jung and Heo [81]	2018	DCNN	SRE loss	Employ SRE loss function
Nguyen et al. [74]	2018	SqueezeNet	Binary CE loss	Optimized lightweight SqueezeNet
Chugh and Jain [26]	2019	DCNN	Binary CE loss	Universal Material Generator
Park et al. [77]	2019	SqueezeNet	Three class CE loss	Tiny and low-cost network
Yuan et al. [82]	2019	DCNN	Binary CE loss	Image Scale Equalization (ISE) layer
Zhang et al. [83]	2019	ResNet	Binary CE loss	Slim-ResCNN framework
Zhang et al. [84]	2020	DenseNet	Binary CE loss	Lightweight FLDNet
Jian et al. [85]	2020	DenseNet	Binary CE loss	Genetic algorithm on DenseNet
Liu et al. [86]	2021	MobileNet V3	Binary CE loss	Rethinking strategy
Rai et al. [87]	2023	MobileNet V1	Support Vector Classifier	Feature extraction and SVC
Grosz et al. [88]	2023	Vision transformer	MSE loss	Joint model for matching and detection
Raja et al. [89]	2023	Vision transformer	Binary CE loss	DeiT-base model
Yuan et al. [90]	2024	Attention residual CNN	Custom loss	Siamese attention residual network
Cheniti et al. [91]	2025	VGG16 and ResNet50	Binary CE loss	A dual-pre-trained design

Image enhancement before detection has been well-studied in deep-learning-based FPAD. Jang et al. [79] utilized histogram equalization for contrast enhancement to improve the recognition rate of fingerprint images. The fingerprint image is divided into several non-overlapped blocks and trained with a CNN model for classification. The Majority Voting System (MVS) is applied to total the votes of all sub-blocks and make the final decision. Similarly, Lazimul and Binoy [78] propose enhancing a fingerprint image through six steps: Image Segmentation, Image Local Normalization, Orientation Estimation, Ridge frequency Estimation, Gabor Filtering, and Image Binarization/thinning. Subsequently, a CNN model is used to train the data. Typically, cross-entropy is the most common loss function used to measure the difference between two probability distributions and is widely applied in classification tasks. Pala [80] introduces a triplet loss [92] that encourages dissimilar pairs to be distant from any similar pair by at least a certain margin value. A triplet network takes two patches of one class and one patch of the other to measure the inter- and intra-class distances supervised by triplet loss. Furthermore, Jung and Heo [81] introduce a new CNN architecture that employs a Squared Regression Error (SRE) layer instead of a cross-entropy loss layer. This method allows for setting a threshold as a liveness probability to adjust the model, which provides an accuracy trade-off option to fit different application scenarios. Furthermore, Jung et al. [93] extend their previous work by introducing two CNNs termed the Liveness Map CNN (LM-CNN) and the Template-Probe CNN (TP-CNN). The LM-CNN performs pre-computation during fingerprint registration to map the fingerprint image to a 32×32 feature map. Then, the output map from the probe fingerprint and template fingerprint is stacked as a 2×32×32 liveness map, which is fed into the TP-CNN for the final decision. Most CNN models require a fixed length of input images because of the restriction of the fully connected layer. Thus, the fingerprint dataset requires additional preprocessing such as cropping or scaling, which leads to information loss. To address this problem, Yuan et al. [82] propose an improved DCNN model with Image Scale Equalization (ISE) to preserve texture information and maintain image resolution. Between the last convolutional layer and the fully connected layer, an extra ISE layer is added to obtain the feature map from the convolutional layer and convert the image of any scale into a fixed-length vector to fix the fully connected layer.

Furthermore, Yuan et al. [94] first introduced the Deep Residual Network [62] for FPAD. The authors designed a novel ROI extraction technique to remove the noise caused by background noise. Then, the gradient disappearance in the DCNN and learning parameters falling into local optimal value issues are tackled by applying a Deep Residual Network with adaptive learning. Owing to the concern of potential low generalization capability against unknown attack detection, a texture enhancement based on a Local Gradient Pattern (LGP) is introduced to highlight the difference between a bona fide sample and an attack sample to achieve a better generalization. Zhang et al. [83] proposed a lightweight framework that makes use of the specifically designed robust Residual block [62] against fingerprint spoofing. Slim-ResCNN consists of nine modified residual blocks. The authors make some changes by inserting a dropout layer into each pair of convolutional layers and removing the activation function (ReLU) of the second convolutional kernel to make the model more generalized. In another specific type, the 1×1 convolutional layer is replaced with max pooling, along with a padding zero channel. Therefore, the overall structure of The Slim-ResCNN consists of Conv1, Conv2, Conv3 (Conv3_1, Conv3_2), and Conv4 (Conv4_1, Conv4_2), followed by global average pooling (Avg_Pool) and a final classification layer. The model will take the extracted local patches as input and the cross-entropy is used as the loss function. It should be noted that this method achieved the top performance in the Fingerprint Liveness Detection Competition 2017 [37], with an overall accuracy of 95.25%. Zhang et al. [84] discussed the limitations of Global Average Pooling against fingerprint spoofing and overcame it by adopting the attention mechanism. A lightweight model with only 0.48 million parameters was designed. Its blocks were designed such that the residual path and densely connected path are incorporated, so the design benefits from DenseNet [63] and ResNet [62]. Jian et al. [85] pointed out the limitations of DenseNet-based architecture [84] and optimized the model by adopting the genetic algorithm [95]. Liu et al. [86] proposed a framework based on the rethinking strategy. The model consists of three modules, a global PAD module, a rethinking module, and a local PAD module. Firstly, the global PAD module receives the entire image as input and then predicts the global spoofness score. The rethinking module then takes the activation map to highlight the important regions for PAD through class activation mapping (CAM). Finally, these regions will be cropped and passed into the local PAD module to refine the prediction of the global PAD module. Rai et al. [87] adopted MobileNet V1 as a feature extractor due to the capacity of utilizing a depth-wise separable convolution operation instead of a traditional convolution operation; then, the network is trained by the loss obtained from SVC. Furthermore, a comprehensive comparison among many existing approaches indicates that the proposed method, namely MoSFPAD, achieved state-of-the-art results. Yuan et al. [90] targeted a specific live–spoof cue: ridge continuity features (RCFs). The authors enhanced ridge textures with Gabor filtering and used an attention Res-CNN to extract RCF-related features. They also introduced a custom ridge continuity amplification loss to emphasize RCF differences and utilize Grad-CAM heatmaps to visualize where the network is focusing. Recently, Cheniti et al. [91] discussed the fact that single-backbone feature extractors may generalize poorly across spoof materials/sensors. The authors proposed a simple but effective dual-pre-trained design by combining the high-resolution features extracted from VGG 16 and deeper representations extracted from ResNet 50. The method achieved strong accuracy on LivDet2013 and LivDet2015, outperforming several baseline approaches.

#### 5.3.2. FPAD Using Transfer Learning/Fine-Tuning

However, end-to-end deep learning-based FPAD achieves a notable improvement in classification accuracy. The size of the public fingerprint training set is insufficient to optimize a CNN model, which typically requires a large number of samples for training. On the one hand, many researchers include data augmentation to apply small variations to the original data to extend the dataset. On the other hand, transfer learning and fine-tuning are normal ways to tackle the issue of small datasets. As shown in Figure 6, transfer learning/fine-tuning is a technique that does not train a deep learning model from scratch. Instead, transfer learning uses the representations learned by a pre-trained model to extract meaningful features and outperform classification with a new classifier. In contrast, the fine-tuning technique is used to unfreeze the weights corresponding to the top few layers of a pre-trained model based on general sets of images and “fine-tune” the higher-order feature to make them more relevant for the specific task. Table 7 presents a quick overview of the existing transfer-learning-based FPAD.

Nogueira et al. [96] extend their work [66] by utilizing transfer learning on a pre-trained VGG [61] and AlexNet [60] model and fine-tuning it using a fingerprint liveness detection dataset. By comparing four different models (two CNN models pre-trained with natural images and fine-tuned with fingerprint images, one CNN-Random model that uses only random filter weights drawn from a Gaussian distribution, and a traditional LBP-SVM model), the authors elaborate on the superiority of pre-trained CNNs on FPAD fields. Furthermore, Toosi et al. [97] extract a set of small-sized patches that contain foreground pixels only, and pass those patches into a pre-trained AlexNet [60] with a further training step that exploits features from fingerprint datasets. Similarly, Toosi et al. [98] extract small-sized foreground patches of raw images and fine-tuned pre-trained AlexNet [60] and VGG19 [61] models. Similarly, Ametefe et al. [99] utilize transfer learning using DenseNet (DenseNet201) [63], which also achieves promising results compared with VGG and AlexNet features.

#### 5.3.3. Generalized Deep Learning

To improve the generalization capacity of the model, many researchers have considered applying a generalized model to transfer one domain to another using an adversarial learning-based model. GANs and style/translation methods expose the classifier to material/style variability, improving robustness to unseen PAIs by narrowing domain shift across sensors and materials. Table 8 presents a quick overview of existing generalizable FPAD techniques.

Pereira et al. [101] proposed a novel model based on adversarial training, which consists of three subnetworks: (i) an encoder network that maps the input image into a latent space, (ii) a task-classifier network that maps the latent representation to the corresponding attack and bona fide probabilities, and (iii) a species-classifier network that aims to predict the PAI species according to the attack latent representation. The species classifier is trained to minimize classification loss among the PAI species, whereas the task classifier and encoder are trained to minimize the classification loss between attacks and bona fide samples while trying to keep the PAI species classification close to random guessing. To further improve the generalization performance of the detector against spoofs made from materials that were not seen during training, a style-transfer-based wrapper, namely, a Universal Material Generator (UMG) is proposed to reliably detect the FPAD [102]. The UMG is able to generate synthetic spoof images corresponding to unknown spoof materials by transferring the style (texture) characteristics between fingerprint images of known spoofing materials. Then, the synthesized images provide the model with more generalization capability to detect spoofs made of unknown materials. Sandouka et al. [103] propose a Unified Generative Adversarial Network (UGAN) that can translate a single generator learning mapping across multiple domains. Subsequently, a share-weighted fusion layer acts as a classifier that fuses the outputs from all translated domains to determine the detection result. Similarly, Sandouka et al. [104] further utilize a CycleGAN [105] network for domain adoption, which transforms the source domain to the target domain. In contrast to their previous work, a transformer model is employed to take a sequence of patches of the feature map as the input. The outputs are then concatenated and projected linearly to obtain a final output that is further fed into a fully connected layer for the classification task. This work further improved the performance compared to that in ref. [103]. Furthermore, Lee et al. [106] proposed a generalization improvement method that utilizes style transfer to transfer the material styles between fingerprints. Liu et al. [107] recently proposed a channel-wise feature denoising model. They extract the ‘noise’ channels from the feature map by evaluating each channel of the image. Then, the interference from those channels is suppressed, and a PA-adaptation loss is designed to align the feature domain of the fingerprint. This method achieves promising results on the LivDet 2017 [37] dataset.

Domain generalization and related robustness-oriented methods are summarized in Table 8 and are referenced in the security-centric discussion on evolving PAIs.

**Table 8:** Existing generalized deep learning FPAD methods.

Author	Year	Backbone	Loss Function	Main Contribution
Pereira et al. [101]	2020	Species-invariant network	Adversarial loss	Adversarial learning
Chugh and Jain [102]	2020	Universal Material Generator	Adversarial and style loss	Style transfer-based approach
Sandouka et al. [103]	2021	GAN, EfficientNet V2	Adversarial loss, reconstruction loss	Unified GAN
Sandouka et al. [104]	2021	Transformer, CycleGAN	Adversarial loss, cycle consistency loss	Domain transfer
Lee et al. [106]	2022	CNN, CycleGAN	Adversarial loss, binary CE loss	Style transfer
Liu et al. [107]	2022	MobileNet V2	PA-Adaptation loss, binary CE loss	Feature denoising model
Anshul et al. [108]	2023	Auxiliary Classifier GAN	Adversarial loss	Enhanced GAN
Rai et al. [109]	2024	GAN	Adversarial loss	Open Patch Generator

### 5.4. Contactless-Based FPAD

Compared to contact sensors, contactless and smartphone acquisitions exhibit larger variations in scale, perspective distortion, illumination, and background clutter. As a result, architectures that incorporate strong geometric/data augmentation, multi-scale features, and domain-generalization objectives tend to perform more reliably under cross-device deployment. The survey therefore highlights protocols that stress *cross-sensor* and *cross-material* generalization and cautions that purely intra-dataset benchmarks can overestimate real-world robustness.

**Figure 7:**
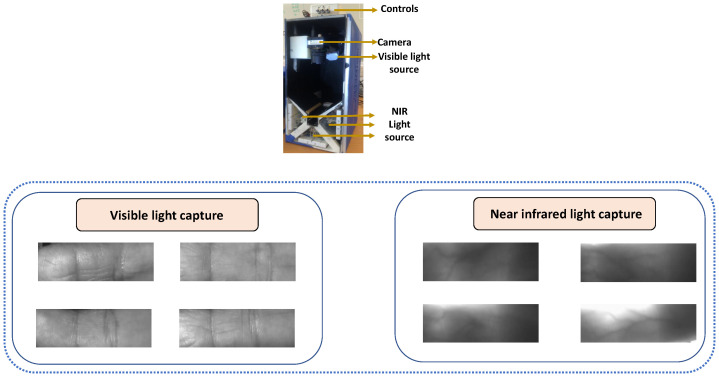
Examples of a contactless-based fingerprint system using sensor-specific approaches.

Figure 7 provides illustrative examples of contactless fingerprint acquisition and associated imaging challenges that motivate robust cross-device FPAD.

Traditional contact-based FPAD primarily relies on texture and subsurface signals captured by touch sensors, whereas contactless FPAD can exploit richer multispectral/3D cues and temporal dynamics. This difference affects modeling choices and the kinds of generalization challenges observed. Contact-based fingerprint images suffer from presentation attacks since the texture features of spoofing are typically inconspicuous. Additionally, the development of new synthetic materials brings more challenges to the generalization ability of the current models. However, the texture of the spoofing fingerprint surface can be visible through a multi-spectrum capture device or even a smartphone camera. A demonstration of a multi-spectrum capture device is shown in Figure 7. Hussein et al. [110] propose a patch-based CNN model that takes multispectral short-wave infrared (SWIR) imaging and laser speckle contrast imaging (LSCI) images as input to classify the images as skin or no skin. Furthermore, Mirzaalian et al. [111] utilize LSCI images and evaluate four different models: the model proposed by [110] terms as baseN, adding residual connections between every two 2D convolution layers of the BaseN terms as ResN, introducing inception module terms as IncpN, and a double-layer long short-term memory (LSTM)-based network. Contactless modalities (e.g., LSCI/SWIR sequences) encode time-varying perfusion/speckle signals linked to liveness; recurrent models can aggregate these temporal cues for improved PAD. The obtained result shows that the LSTM-based approach achieves the best performance. Kolberg et al. [112] analyze the long short-term memory (LSTM) network in comparison with different CNN models on a fingerprint image captured by a 1310 nm laser device. The obtained experimental results indicate the advancement of the LSTM model compared with the CNN models. Furthermore, Spinoulas et al. [113] evaluate FPAD performance under different sensing modalities using a fully convolutional neural network (FCN). The evaluation experiments are carried out under fingerprint images captured from Visible (VIS), near-infrared (NIR), SWIR, LSCI and near-infrared back-illumination domains.

Table 9 is referenced to compare contactless FPAD methods under challenging acquisition variability.

#### Anomaly Detection

**Figure 8:**
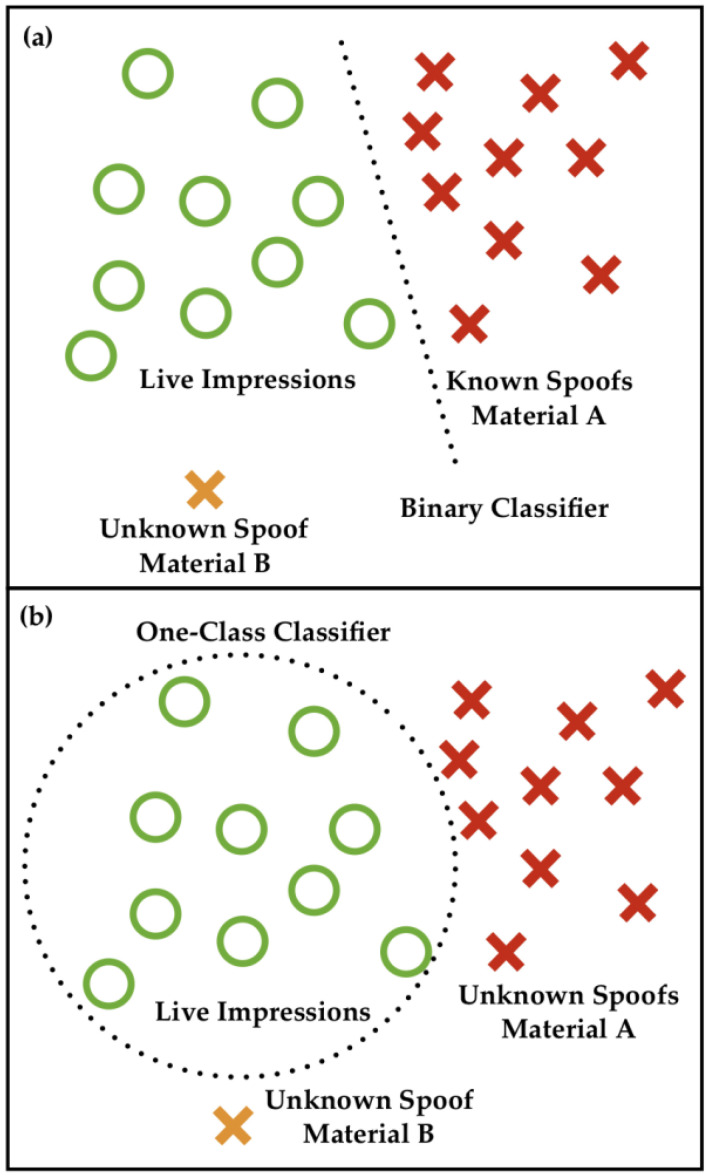
An example of binary classifier (**a**) and anomaly detection (**b**).

Most previous deep learning models formulate FPAD as a close-set problem to detect various predefined PAs, which require large-scale training data to cover as many attacks as possible. In addition, the training data must be labeled prior to training. However, this leads to an overfitting issue in that the model is highly sensitive to the PAs already included in the training dataset but lacks generalization capability against unseen attacks. An increasing number of novel materials have been developed to produce gummy fingers to deceive FRS easily [116]. The unknown FPAD method has become an open issue and has attracted increasing attention in recent years. Compared with the most common binary classifier, the one-class classifier only learns the representation of a live fingerprint and does not use spoof impressions of any specific material. Then, the unseen attack is detected as an anomaly, which is performed as an outlier compared with the bona fide samples. Figure 8 shows the difference between the binary classifier and anomaly detection-based approaches.

Shown in Table 10, Engelsma and Jain [117] propose a fingerprint spoof detector on only live fingerprints by training multiple generative adversarial networks (GANS) on live fingerprint images. Three GAN models are trained on raw FTIR images from RaspiReader, processed FTIR images, and direct-view images. For each GAN, the generator attempts to synthesize live fingerprint images, where the discriminator uses the generated samples as well as the true samples from the dataset to distinguish them. Thus, through long iteration training, the generator is trained to generate high-quality images where the discriminator is able to separate the liveness sample from the generated sample. After the training phase, the generator is discarded, and the discriminator can be used as an FPAD module to detect attacks. Finally, fusion of the scores output by all three discriminators constitutes the final spoofness score of an input fingerprint sample. Rohrer and Kolberg [118] first utilize the Wasserstein GAN as a pre-trained model trained with the LivDet2021 [39] Dermalog Sensor dataset from scratch. The GANs discriminator weights are transferred to the AutoEncoder’s (AE) encoder, whereas the generator weights are transferred to the decoder. A convolutional layer is added between the encoder and decoder to connect them. The AE learns to minimize reconstruction loss [119] so that the model can reconstruct the input image with minimal reconstruction error. The PA will be detected with a large reconstruction error.

Kolberg et al. [120] propose a new PAD technique based on autoencoders (AEs) trained only on bona fide samples captured in the short-wave infrared domain, which converts the two-class classification problem into a one-class domain. The authors introduce three AE architectures for Conv-AE, Pooling-AE, and Dense-AE, and compare the results with other state-of-the-art one-class PAD. In addition, Liu et al. [121] propose a novel One-Class PAD (OCPAD) method for Optical Coherence Technology (OCT) images that provides an internal representation of the fingertip skin rather than a simple feature. The proposed PAD framework consists of auto-encoder network-based reference bona fide modeling and spoofness score-based PA detection. Furthermore, Liu et al. [122] modify the autoencoder-based model by introducing a prototype memory module. In the training phase, the denoising autoencoder is optimized by minimizing the reconstruction error. The memory module will update the recording of latent representation output by the encoder.

### 5.5. Smartphone-Based FPAD

The rapid development of smartphone-based authentication applications has achieved high verification accuracy [123], which has raised concerns about the smartphone-based system being easily spoofed. Zhang et al. [124] proposes a 2D smartphone-based approach that combines the features of two local descriptors (LBP and LPQ) with deep features extracted from a CNN model. The CNN model is optimized by integrating global average pooling and batch normalization. Due to the lack of publicly available datasets, self-obtained bona fide samples and 2D attack samples made of wood glue, electrosol from PCB, or printed by special conductive ink are built. By fusing the results of the two descriptors and the CNN, the final decision can be output. Fujio et al. [125] compare the performance of using a handcrafted-based method (LBP, LPQ) and a deep learning-based method. The obtained results indicate that the DCNN (AlexNet) can achieve a stable accuracy when the intensity of the blurring noise increases.

Marasco and Vurity [126] explored detection performance by training the IIITD database using various CNN architectures (AlexNet [60] and ResNet18 [62]). The comparison results demonstrate that AlexNet achieved a robust performance against unseen attacks. The authors further propose a novel method [127] that explores the detection effectiveness of different CNN models on different color spaces. The raw image is converted into RGB, YCBCr, HSV, LAB, and XYZ color spaces, and the five images are then further fed into five pre-trained CNN models (AlexNet [60], DenseNet201 [63], DenseNet121, ResNet18 [62], ResNet34, and MobileNet-V2 [73]). The best network is selected, and the score of the five color spaces is fused to obtain the final decision. Recently, to address the lack of publicly available fingerphoto presentation attack detection datasets, Purnapatra et al. [44] proposed a new dataset comprising six different PAIs. The FPAD methods employed use the state-of-the-art CNN models DenseNet 121 and NASNet [128], which achieve promising PAD accuracy on the proposed dataset. Li and Raghavendra et al. [129] compared eight different deep feature extraction models and train the SVM model to classify the bona fide and attack data. In [130], the authors further analyzed and discussed the effects of different image segmentation approaches (original image, ROI image, and background removal image) using a scheme similar to that in [129]. The obtained results indicate that the fingerphoto background significantly affects detection performance to a large extent. Adami et al. [131] introduced a universal semi-supervised method based on ResNet18 model with different activation functions and jointly supervised by arcFace loss and center loss. The model was trained using bona fide fingerphoto samples and synthetic data generated by StyleGAN-ada [132]. The obtained results indicate a good generalization ability against unseen attacks. Recently, Priesnitz et al. [133] focused on the generalizability and explainability aspects of fingerphoto PAD; they selected four different PAD methods designed for different biometric characteristics and benchmarked the evaluation performance among four different database using the Leave-One-Out (LOO) protocol. Additionally, a T-SNE plot was applied to visually interpret the obtained results. The LOO evaluation obtained a low D-EER with below 0.1%, but the cross-database evaluation achieved a D-EER of 4.14%.

However, most of the prior fingerphoto PAD methods are supervised methods with poor generalizability against unseen attacks. Liu et al. [134] proposed an unsupervised approach utilizing a Memory-augmented Autoencoder that trained solely on bona fide samples. The spoof score was calculated by MSE between the input and output features. Similarly, Adami et al. [135] presented an unsupervised autoencoder + attention scheme trained solely on bona fide contactless fingerprints and tested against diverse spoof types, demonstrating low error rates and highlighting the viability of bona fide-only training for fingerphoto PAD. Furthermore, Li et al. [136] also tried to tackle fingerphoto PAD in an open-set setting by training a DDPM only on bona fide images and flagging attacks via input–output reconstruction similarity. The authors evaluated across multiple PAI datasets, and the method showed improved generalization to never-seen attacks compared with other unsupervised baselines. Recently, Adami and Karimian proposed a Swin-UNet backbone enhanced with GRU attention using a Dynamic Filter bottleneck and focal+contrastive losses to align domains across contactless sensors, achieving strong cross-dataset results. Lastly, Li et al. [137] explored the potential of LLMs as generalizable and explainable PAD solutions, demonstrating the feasibility of detecting fingerphoto presentation attacks using multimodal large language models.

Smartphone/fingerphoto FPAD methods are summarized in Table 11 to highlight mobile deployment constraints.

**Table 11:** Existing state-of-the-art smartphone-based FPAD detection methods.

Author	Year	Backbone	Loss Function	Main Contribution
Zhang et al. [124]	2016	CNN	Binary CE loss	Handcrafted features and SVM
Fujio et al. [125]	2018	AlexNet	Binary CE loss	CNN-based method
Marasco and Vurity [126]	2021	AlexNet, ResNet18	Binary CE loss	Evaluate different CNNs
Marasco et al. [127]	2022	6 CNN models	Binary CE loss	Explore various color spaces
Purnapatra et al. [44]	2023	DenseNet 121 and NASNet	Binary CE loss	A new fingerphoto dataset
Li and Raghavendra [129]	2023	8 CNN models	SVM	Compare 8 CNNs
Adami et al. [131]	2023	ResNet18	Arcface loss and centre loss	Semi-supervised approach
Priesnitz et al. [138]	2023	9 different models	Binary CE loss	Benchmark various models
Li and Raghavendra [130]	2024	8 CNN models	SVM	Apply different preprocessing strategy
Priesnitz et al. [133]	2024	4 CNN models	Binary CE loss	Explore generalizability and explainability ability
Liu et al. [134]	2024	Autoencoder	MSE loss	An unsupervised approach
Li et al. [136]	2024	Diffusion	MSE loss	one-class approach
Adami et al. [135]	2024	Autoencoder	MSE loss	An unsupervised approach
Vurity et al. [139]	2025	MobileNet-V3	Binary CE loss	Multiple color spaces
Adami and Karimian [140]	2025	Swin-UNet	Binary CE loss	Domain adaptation
Li et al. [137]	2025	LLM	NA	Attempt to use LLM for PAD

### 5.6. FPAD Using Hybrid Feature Extraction Methods

The hybrid method refers to combining more than one type of feature (handcrafted features, deep features, and multi-spectrum features, etc.) to detect the PAIs. The hybrid features can be used with all types of fingerprint capture devices and have been demonstrated to achieve higher detection accuracy at the cost of computation. Table 12 briefly discusses the state-of-the-art hybrid FPAD methods.

Jomaa et al. [141] utilize electrocardiogram (ECG) signals as well as deep features to jointly make decisions. Furthermore, Tolosana et al. [142] propose a multi-model-based approach that utilizes four deep features extracted, respectively, from a five-layer residual network, a pre-trained Mobile-Net-based network, a pre-trained VGG 19-based network, and a CNN model, and combined the deep features with a spectral signal from a short-wave infrared (SWIR) spectrum capture device to jointly determine the liveness. Gomez et al. [143] analyze the surface of a finger within the SWIR spectrum and inside the finger using laser speckle contrast imaging (LSCI) technology. The SWIR feature is extracted by the ResNet and VGG19 network combined with the handcrafted feature extracted from the LSCI using BSIF, HOG, and LBP descriptors. Then, the fusion of the results obtained from the above techniques determines the final liveness score. Plesh et al. [144] propose a novel approach that combines dynamic time-series features with static features extracted by the Inception-V3 [72] CNN model. Then, the final result is obtained by the fusion of two feature sets. The experiments demonstrated that the fusion of two feature sets achieves a better performance than the individual features. Kolberg et al. [145] also include SWIR and laser techniques to analyze spoofing. In contrast to [143], they select a long-term recurrent convolutional network (LRCN) [146], a pre-trained CNN model, and an autoencoder network to independently obtain a liveness score from the laser image. Meanwhile, the CNN and autoencoder models process the image from the SWIR. Finally, subsequent score-fusion is applied to obtain the final score for classification.

Hybrid FPAD approaches are summarized in Table 12 and are referenced when discussing multi-cue robustness.

### 5.7. Practical Deployment Considerations

In operational biometric systems, FPAD is typically integrated either *serially* (PAD first, then matcher) or *in parallel* (PAD score fused with matching score), as illustrated by the system pipeline in Figure 3. Key implementation constraints include (i) real-time latency budgets (e.g., mobile unlock vs. border control), (ii) compute/memory limits for on-device inference, (iii) calibration of operating points to balance security and usability, and (iv) update strategies to handle evolving PAI materials and sensor upgrades. Accordingly, the manuscript now highlights, when reported by the original authors, model size, inference cost, and whether the method supports lightweight deployment (quantization, pruning, or compact backbones).

**Table 12:** State-of-the-art hybrid FPAD methods.

Author	Year	Backbone	Loss Function	Main Contribution
Tolosana et al. [142]	2018	RenNet, MobileNet, VGG19	Binary CE loss	Combine deep and SWIR features
Gomez et al. [143]	2019	ResNet and VGG	Binary CE loss	Combine LSCI and SWIR features
Plesh et al. [144]	2019	Inception-V3	Binary CE loss	Combine time-series feature with deep feature
Jomaa et al. [141]	2020	MobileNet-v2	Binary CE loss	Combine ECG feature and deep feature
Kolberg et al. [145]	2021	LRCN, CNN and AutoEncoder	Binary CE loss	Combine laser and SWIR features

## 6. Interpretability of Fingerprint Presentation Attack Detection

Despite the high performance of the majority of existing deep learning-based FPAD approaches, it is still important and necessary to include an explainable solution to promote end-user trust, model auditability, and productive use of AI. Explainable Artificial Intelligence (XAI) refers to the illumination of what is going on in the “black box” that often surrounds AI’s inner workings. However, in recent studies, many researchers have included XAI methods to make models transparent.

Beyond simple visualizations, explainability is most useful in FPAD when it (i) confirms that the detector relies on physically plausible cues (ridge continuity, pore-level texture, specular highlights, or print artifacts) rather than sensor- or background-specific shortcuts, and (ii) supports *attack forensics* by localizing which regions are indicative of a particular PAI material or fabrication defect. Accordingly, the survey now compares common post hoc tools and discusses their relative strengths: activation-map methods are intuitive but can be unstable under strong domain shift, while perturbation-based methods are often more faithful but computationally more expensive. Importantly, interpretability is linked to deployment and certification contexts, where auditors may require evidence of consistent decision rationale across sensors and demographic cohorts, and where explanation artifacts can be logged to support incident analysis following suspected presentation attacks.

Dastagiri et al. [147] propose an attentive interpretable deep learning model that is able to output the predicted class (live or fake) and feature importance for each instance encountered to the trained model. Yuan et al. [90] leverage Gradient-weighted Class Activation Mapping (Grad-CAM) [148] to generate a heatmap that visualizes the interpretability of their proposed Siamese attention residual convolutional neural network. Especially when evaluating the cross-material scenario, the heatmap highlights regions where the continuity of fake fingerprint ridges is poor. More works, such as [107,149], also plot the heatmap to help understand the decision made by the model. Table 13 lists the methods that used interpretation tools.

## 7. Performance Evaluation Metrics

In this section, we discuss different evaluation metrics that are widely used in the fingerprint PAD literature. First, we present the metrics used in LivDet competitions [33], followed by ISO/IEC using ISO/IEC 30107-3 [150] metrics.

### 7.1. Evaluation Metrics from LivDet Competitions

Since the first edition of the Fingerprint Liveness Detection Competition (LivDet) in 2009 [33], the following performance evaluation metrics have been used to benchmark the performance of the FPAD algorithms:

- **Frej**: Rate of failure to enroll. *Failure to enroll* indicates inability to extract features from the fingerprints of certain individual.
- **Fcorrlive**: Rate of the live fingerprint to be classified correctly.
- **Fcorrfake**: Rate of the fake fingerprint to be classified correctly.
- **Ferrlive**: Rate of the live fingerprint to be misclassified.
- **Ferrfake**: Rate of the fake fingerprint to be misclassified.

Additional evaluation metrics such as average classification error (ACE) are defined in [71]:

(1) $$ACE=\frac{Fcorrlive+Fcorrfake}{2}$$

Starting from LivDet 2021 competition, evaluation metrics are defined according to the ISO/IEC 30107–1 standard presented below.

### 7.2. ISO/IEC Metrics for PAD

The International Standard Organization (ISO/IEC 30107–1:2016) [151] has described the general framework to present the attack detection performance results. The ISO/IEC 30107–1 framework defined following metrics:

- **Liveness Accuracy**: Rate of samples correctly classified by the PAD system.
- **APCER (Attack Presentation Classification Error Rate)**: Percentage ratio of presentation attack test samples misidentified as bona fide samples.
- **BPCER (Bona fide Presentation Classification Error Rate)**: Percentage ratio of bona fide test samples misidentified as presentation attack samples.

**D-EER (Detection Equal Error Rate)** indicates the point where the BPCER equals the APCER in a biometric system. Normally, D-EER is a key operating point that summarizes system performance. The lower the value, the better the system. To further evaluate the performance of the integrated system, additional metrics are also defined:

- **FNMR (False Non-Match Rate)**: Rate of genuine fingerprints to be classified as an impostor.
- **FMR (False Match Rate)**: Rate of zero-effort impostors classified as genuine.
- **IAPMR (Impostor Attack Presentation Match Rate)**: Rate of impostor attack presentations classified as genuine.
- **Integrated Matching Accuracy**: Rate of samples correctly classified by the integrated system.

### 7.3. Critical Discussion of Metrics and Benchmarking Bias

While APCER/BPCER/ACE are widely adopted, their interpretation can be unstable under class imbalance, evolving PAIs, and dataset-specific priors. For example, a small number of unseen PAI materials can dominate the operational risk even when average ACE appears low. Therefore, the manuscript now recommends reporting: (i) operating-point curves with confidence intervals, (ii) per-material APCER, and (iii) cross-sensor/cross-material splits that reflect deployment. In addition, benchmark protocols such as LivDet may inadvertently favor methods that exploit sensor-specific artifacts; thus, subject-disjoint and device-disjoint evaluations, along with leakage checks, are emphasized. Finally, composite or application-driven metrics are suggested for security-centric decision making rather than relying on a single scalar score.

### 7.4. Benchmark

To enable cross-method comparison, we consolidate the benchmark results reported in the literature and summarize them in Table 14 and Table 15. However, direct cross-paper comparison in FPAD is challenging because studies often use different datasets, train/test splits, and even different reporting metrics. Therefore, we adopt a transparent cross-paper normalization policy when compiling the benchmarks: (i) we prioritize ISO/IEC-style operating-point metrics (APCER/BPCER) and ACE when available. (ii) If only accuracy is reported, we keep it as-is and treat it as not directly comparable to ACE/APCER/BPCER because the decision threshold and class priors are typically unspecified. (iii) We avoid averaging results across different protocols and instead retain the protocol context alongside each value, so the tables serve as an evidence map rather than an over-interpreted league table.

In addition, to reflect practical deployment constraints, we include a computation label (Low/Medium/High) that estimates inference burden primarily from the backbone family (e.g., MobileNet/SqueezeNet as low, compact CNNs such as ResNet18 as medium, and larger backbones such as Inception/ViT or GAN-based pipelines as high). Importantly, higher computation does not consistently translate into better security: lightweight backbones can achieve competitive in-domain results, while heavier models may still fail under domain shift (new sensors, materials, or acquisition conditions). For this reason, we emphasize that future benchmarking should report at minimum latency, parameter count, and the input/patching strategy to make cost–accuracy trade-offs operationally meaningful.

Finally, reproducibility and dataset leakage are critical when interpreting benchmark numbers. FPAD is particularly vulnerable to hidden leakage through (i) subject/session overlap between train and test sets, (ii) patch-based pipelines where patches originating from the same fingerprint impression can appear in both splits, and (iii) repeated tuning on public test sets. When consolidating results, we prioritize studies that clearly specify split strategy and provide sufficient implementation details. We recommend that benchmark reporting explicitly state the disjointness level and contamination checks, since these factors can inflate reported performance without improving real-world robustness.

#### 7.4.1. Contact-Based FPAD Method Benchmark

For contact-based FPAD approaches, we covered the methods for end–end DL-based approaches, transfer learning/fine-tuning-based approaches and generalized deep learning approaches. Those methods are evaluated on the LivDet public dataset, which is straightforward for comparison between methods. The benchmark can be found in Table 14.

#### 7.4.2. Smartphone FPAD Method Benchmark

For smartphone-based FPAD methods, besides the authors’ own evaluation, we select five original methods and evaluate using our own protocol and selected dataset (CLARKSON, NTNU and a self-collected dataset). We evaluate them using the Leave-One-Out protocol, which selects one dataset as a test set and trains with the other two datasets to simulate the unseen attack scenario. The benchmark of Fingerphoto PAD methods can be found in Table 15.

## 8. Future Work

**Figure 9:**
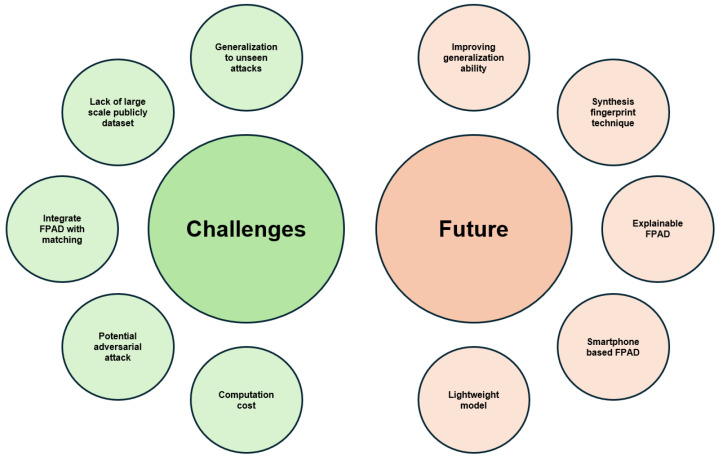
Future Perspective and challenges of FPAD techniques.

With the revolution of deep learning, training deep neural networks (DNNs) has dominated the field of image classification and object recognition. This technique was further extended to FPAD methods, and achieved notable improvements in the detection of fabricated fingerprint replicas. However, some limitations of this study need to be considered and discussed. In this section, we introduce the major challenges of current research and future perspectives. Figure 9 demonstrates the current challenges and the potential future work.

### 8.1. Generalization to Unknown Attack Detection

Normally, a deep learning-based FPAD model takes both bona fide and attack samples for training so that the classifier can distinguish liveness based on the probability score calculated corresponding to the labels. However, these methods suffer from low generalization ability against PAIs not included in the training set. Empirically, FPAD models degrade when deployed on new sensors/spectra (FTIR→SWIR/LSCI/fingerphoto), new PAI materials, and altered capture protocols. These induce (1) marginal and (2) conditional distribution shifts—often stronger for spoof than live—that invalidate decision boundaries learned in-domain. Models trained with limited material diversity can overfit to surface textures, while contactless temporal signals demand different inductive biases. The mitigation space thus includes domain generalization/adaptation, synthetic PAI/style augmentation, and one-class learning that models the bona fide manifold.

The anomaly detection-based approach trains a one-class classifier based on only the bona fide samples to better represent real fingerprint images to detect anomalies that achieve acceptable results against unknown attacks to some extent. In [103], the authors proposed a domain-adaptation approach that can generate mappings across sensors to reduce the distribution shift between different fingerprint representations. Based on this approach as a starting point, it is worth exploring how to make the model learn the mapping from a source domain to an unseen domain to achieve a general representation of the fake fingerprint.

To reduce reliance on large labeled FPAD datasets and improve robustness to new sensors and PAIs, future work should explore self-supervised pre-training (e.g., contrastive or masked-image objectives) on pooled multi-sensor data, followed by light supervised fine-tuning. These approaches can learn sensor- and material-invariant texture cues (ridges, pores, specular seams) and typically yield better cross-material and cross-sensor transfer than training from scratch.

Promising directions include multi-source domain generalization with style randomization/transfer, adversarial alignment (feature-level domain discriminators), few-shot adaptation via metric-learning prototypes and episodic training, test-time batch-norm adaptation and entropy minimization, and synthetic PAI augmentation using style/texture transfer to cover unknown materials. Prior FPAD works on style transfer and multi-domain translation have already shown gains under cross-material/cross-sensor protocols, suggesting these strategies as strong baselines for future work.

### 8.2. Interpretability to Fingerprint Presentation Attack Detection

The widely deployed AI application raised the interpretability issue of how to make the deep learning model explainable. As described in Section 6, it is important to be able to explain the black box deep model in order to understand their decision. Therefore, it is important to build ’Transparent’ models that have the ability to explain why they predict what they predict. In order to provide an analysis of the interpretation of FPAD methods using visualization, techniques such as [148,152,153] can be used to highlight the region of an image that affects the final decision. Lie et al. [107] include Grad-CAM to visualize the important regions related to the given label. It will be interesting to consider applying visualization, especially to images captured by multi-spectrum devices. In the future, more interpretation tools are expected to be applied to FPAD methods.

### 8.3. Lightweight Models for Fingerphoto Presentation Attack Detection

With the rapid development of smartphone cameras, high-resolution fingerphotos can be captured efficiently and directly from a mobile device to aid reliable biometric authentication. Because mobile devices mostly do not have a high-computation environment, a lightweight deep learning model with fewer parameters and focusing on only a small region of the fingerprint images would be an optimal solution. Hence, another perspective could be the presentation attack detection of smartphone-based approaches.

### 8.4. Lack of a Large-Scale Publicly Available Dataset

As deep learning has shown a significant impact on the image classification domain, research on FPAD methods has concentrated on training a large-scale neural network to detect spoofing. Because of the requirement of large-scale sample datasets for both bona fide and attack samples when using a deep learning model, there have been plenty of publicly available datasets published using different capture devices and spoofing materials. However, datasets comprising a large number of samples are still lacking. Particularly in contactless fingerphotos, there is currently a lack of datasets containing bona fide and spoofing samples. A further perspective could be to produce large-scale finger photo presentation attack datasets comprising various presentation attack instrument materials.

Furthermore, since data sharing is often restricted, federated learning across institutions and devices can broaden domain coverage without centralizing images. Practical variants (e.g., keeping batch-norm statistics local and using lightweight personalized heads) help handle site-specific capture conditions while maintaining a strong global model.

### 8.5. Potential Adversarial Presentation Attack

An adversarial attack generates adversarial samples on purpose in order to mislead the image classification result of a machine learning model. One simple way to generate adversarial examples is to add a perturbation of some pixels so that the output image looks no different from the input image, but the classification result will be changed. Beyond conventional PAIs, adversarial examples exploit model sensitivities. In FPAD, this spans: (i) digital attacks (white-/black-box) against the PAD module; (ii) physical adversarial artifacts (e.g., printed patches or mold perturbations) that survive re-imaging; and (iii) pipeline-level attacks that combine injection and physical spoofs.

Another notable observation was proposed by Casula et al. [154], who produced high-quality spoofs through the snapshot picture of a smartphone to obtain the fingerprint latent. Through digital processing, the spoof was fabricated using a transparent sheet. The experiment indicated that this ScreenSpoof presented a threat at the same level as a normal presentation attack. Hence, Marrone et al. [155] investigated the feasibility of adopting an adversarial attack on a physical domain by materially realizing a fake image based on an adversarial fingerprint example. The evaluation of the attack indicates that printed adversarial images exhibit a high attack rate with multiple attacks and fairly good results with a one-shot attack.

Early studies demonstrate feasibility and transfer across models. Current defenses (adversarial training, gradient obfuscation, pre-processing) improve in-distribution robustness but can degrade cross-domain PAD, highlighting the need for certified defenses, expectation-over-transforms tailored to sensing pipelines, and sensor-aware training. According to this, it should be considered that with adversarial examples targeting the FPAD module, it is possible to combine other digital attacks (Masterprint, morphing, etc.), as well as adversarial perturbation to fool the FPAD system to perform more dangerous attacks on the FRS. Hence, it is interesting to exploring countermeasures against emerging adversarial attacks.

### 8.6. Multimodal Fusion for Fingerprint PAD

In many deployments, fingerprint PAD operates alongside other biometrics modality. Fusing biometrics can improve security (lower attack success under unknown materials/sensors) and availability (graceful fallback when one modality is poor). The fusion strategy can be divided into the following aspects:

- **Decision-level:** Independently calibrate PAD scores per modality, fuse identity via weighted logit-sum or logistic regression only across modalities that pass PAD. Report single-modality vs. fused performance.
- **Score-level:** Multiply match evidence by a PAD-derived weight, and add quality-aware gating using no-reference metrics: blur (face), occlusion/eyelid (iris), moisture/pressure (fingerprint).
- **Feature-level:** Learn a lightweight cross-attention between face/iris embeddings and fingerprint texture features to share presentation-artifact cues (e.g., specular patterns, paper/print periodicity). Use modality-dropout so the system degrades gracefully if one stream is absent.

Cross-biometric fusion can significantly reduce successful presentation attacks, especially when one modality is compromised. The key is per-modality PAD calibration, policy-aligned thresholds, and transparent reporting of fused behavior under single and dual spoof scenarios.

### 8.7. Large Language Models (LLMs) for FPAD

Emerging multimodal LLMs (vision–language models) offer a promising, complementary path to FPAD by treating spoof detection as a reasoning problem rather than a purely discriminative one. Instead of extensive task-specific training, LLMs can be prompted with a few bona fide/attack exemplars and structured checklists to produce a verdict and an accompanying explanation. This zero-/few-shot, prompt-based approach is attractive for generalization, interpretability (textual rationales that can be audited), and data efficiency (reduced reliance on large labeled corpora). In practice, LLM outputs can be (i) calibrated on a small validation split, (ii) fused with lightweight CNN features or classical PAD cues for robustness, and (iii) constrained by abstention rules for low-confidence cases. Future efforts should explore on-device or privacy-preserving deployment (e.g., distilled VLMs, federated prompting), safety against adversarial prompts and visual attacks, and standardized protocols to evaluate cross-domain reliability and explanation quality alongside APCER/BPCER, ACE, and D-EER.

To make this direction more concrete for FPAD, several practical uses are highlighted: (i) *patch-based reasoning*, in which a lightweight CNN proposes salient regions and a multimodal LLM is prompted to produce a structured rationale (e.g., ridge discontinuities, specular highlights, printing artifacts) that can be logged for audit; (ii) *protocol-aware reporting*, in which prompts enforce consistent disclosure of cross-material and cross-sensor splits, operating points (e.g., BPCER@APCER = 1%/5%/10%), per-material APCER (average and worst case), and confidence intervals; (iii) *human-in-the-loop triage*, where borderline samples are routed to an analyst with the model explanation, enabling safer abstention rather than forced decisions; and (iv) *data curation assistance*, where the LLM helps categorize PAI materials, annotate acquisition conditions, and flag suspicious duplicates that may indicate leakage. These uses are positioned as complements to discriminative FPAD models rather than replacements, and future work should emphasize calibrated abstention, rigorous security testing (including prompt- and input-level attacks), and privacy-preserving deployment.

## 9. Discussion

**Figure 10:**
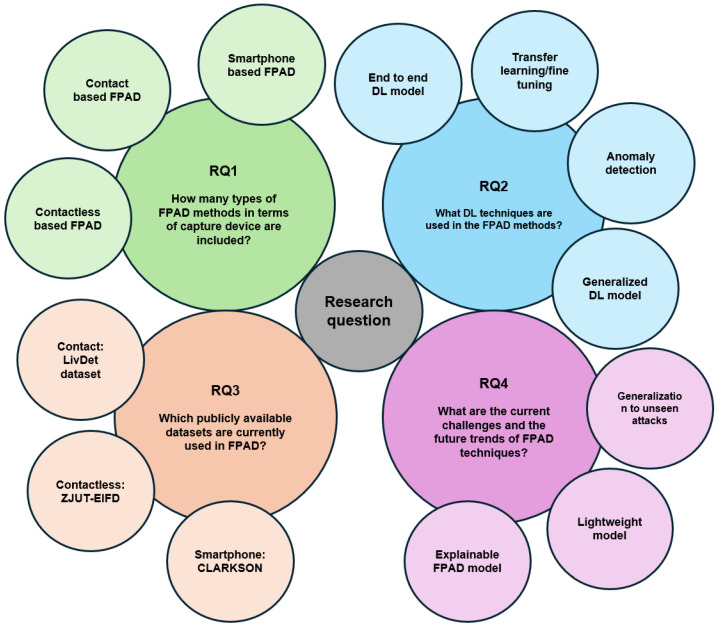
Key items of the answers to the proposed research questions.

To summarize the survey’s guiding structure and contributions, Figure 10 visualizes the main research questions (RQ1–RQ4) and their connections to the reviewed method families and evaluation protocols.

This survey reported in detail the contributions in the domain of deep learning-based fingerprint presentation attack detection methods. Throughout this study, we address the research questions proposed in Section 1. In this section, we provide a summary and discussion of the research questions.

### 9.1. RQ1: How Many Types of FPAD Methods in Terms of Capture Device Are Included?

As shown in Figure 2, we divided FPAD methods into three different types: contact-based, contactless-based and smartphone-based approaches. Contact-based FPAD methods refer to the algorithm deployed on a traditional contact-based capture device. Contactless-based FPAD methods refer to the detection of spoofs based on multi-spectrum devices. Smartphone biometrics has become popular owing to the rapid development of high-resolution cameras. Smartphone-based FPAD refers to the detection of the spoof captured by a smartphone camera.

### 9.2. RQ2: What DL Techniques Are Used in the FPAD Methods?

The evolution of FPAD methods reflects a gradual shift from handcrafted, sensor-specific cues toward data-driven representations designed to operate under increasing variability. Early work mainly relied on texture- and artifact-based descriptors engineered for particular sensors and known PAIs, which provided strong in-domain performance but limited transfer across datasets and acquisition conditions.

A key paradigm shift occurred with the adoption of deep learning, where feature learning replaced manual design and enabled end-to-end optimization. This improved accuracy on large benchmarks, but also revealed new failure modes: sensitivity to domain shift, reliance on spurious dataset cues, and reduced interpretability. In response, more recent research has moved toward *robustness-centric* paradigms, including domain generalization/adaptation, self-/semi-supervised representation learning, and one-class/anomaly detection settings that better match the open-set nature of spoofing.

Overall, the trend is from *closed-set classification* toward *open-set robustness and deployability*, where evaluation across sensors/materials, uncertainty-aware decisions, and efficiency constraints increasingly shape what is considered progress.

### 9.3. RQ3: Which Publicly Available Datasets Are Currently Used in FPAD?

To the best of our knowledge, the publicly available datasets are listed in Table 4. The most famous and widely used contact-based dataset is the Fingerprint Liveness Detection Competition series (from 2009 to 2023). These datasets contain attack samples using different types of PAIs and capture devices, which have been benchmarked by many researchers. ZJUT-EIFD is a large dataset that consists of over 70,000 OCT images of fingerprint-attached samples. Recently, two smartphone fingerprint presentation attack detection datasets, captured by different smartphone cameras, have been released.

### 9.4. RQ4: What Are the Current Challenges and the Future Trends of FPAD Techniques?

The main challenge in FPAD is not only improving performance on a fixed benchmark, but ensuring *reliable operation under change*: new sensors, evolving PAI materials, different acquisition conditions, and attacker adaptation. Section 8 shows that generalizability is therefore a central issue, but it is tightly connected to several other practical and methodological bottlenecks.

First, limited data realism and diversity (PAI coverage, subject/sensor imbalance, and acquisition bias) often leads models to learn dataset-specific cues, which directly increases domain-shift failures. Second, FPAD is inherently open-set: unseen materials and fabrication pipelines appear in practice, so calibrated uncertainty and open-set evaluation are needed beyond closed-set testing. Third, protocol and metric inconsistency across studies (splits, cross-sensor/cross-material settings, and reporting choices) reduces comparability and can overstate progress. Fourth, security robustness must consider both physical and digital threat models, since adaptive attackers can exploit model or pipeline weaknesses. Finally, deployment constraints (latency, compute, and user experience) and the need for explainability affect what methods are trustworthy and adoptable.

These findings suggest future trends that combine robustness and practicality: (i) standardized cross-domain protocols and richer benchmarks; (ii) learning strategies for open-set robustness (e.g., one-class/anomaly detection, domain generalization/adaptation, self-/semi-supervision, and uncertainty-aware decisions); (iii) security-aware evaluation against adaptive attacks; and (iv) efficient and interpretable FPAD suitable for real devices.

## 10. Conclusions

This survey mapped deep-learning FPAD across capture settings (contact, contactless, and smartphone) and learning paradigms, while unifying datasets and evaluation metrics to support clearer comparisons. Although modern CNNs achieve strong in-domain performance on LivDet-style benchmarks, reliability often drops under domain shift from new sensors, materials, or acquisition conditions, reflecting limitations in dataset diversity and reporting consistency. From a deployment perspective, supervised end-to-end CNN detectors remain attractive due to simplicity and high accuracy when the sensor and PAI set are stable, but they may overfit to dataset- or sensor-specific artifacts and become brittle under spoof evolution. Transfer learning can reduce data requirements and accelerate adaptation to new devices, yet it can also suffer from negative transfer when source-domain textures differ from the target sensor. Domain generalization/adaptation and hybrid multi-cue designs can improve cross-sensor robustness, typically at the cost of higher training complexity and occasionally reduced in-domain accuracy. One-class/anomaly-oriented FPAD better matches open-set security requirements, but requires careful calibration to avoid excessive bona fide rejection and usability degradation. Based on these findings, we highlight practical directions for future work: standardized cross-material/cross-sensor protocols, lightweight models for on-device use, data-efficient learning (self-/semi-supervised, few/zero-shot, and one-class), domain adaptation and synthetic PAI augmentation, and interpretable analyses that reveal failure modes for auditing and incident forensics. Finally, multimodal LLMs offer a complementary reasoning-centric path (e.g., zero/few-shot prompting with calibrated abstention) that may be fused with compact CNN features to improve generalization and transparency. Overall, progress should move beyond single-number benchmarks toward operationally meaningful trade-offs evaluated under transparent, security-aware protocols.

## Figures and Tables

**Table 1:** Number of articles extracted from different databases.

Datasource	ScienceDirect	Scopus	arXiv	IEEE Xplore	Total
# articles	11	9	9	38	**67**

**Table 2:** A summary of existing surveys in FPAD.

Paper Title/Reference	Year	Deep Learning Included	Modality and Hardware
Survey on fingerprint liveness detection [3]	2013	No	Contact-based
Presentation attack detection methods for fingerprint recognition systems: a survey [4]	2014	No	Contact-based
A Survey on Antispoofing Schemes for Fingerprint Recognition Systems [5]	2014	No	Contact-based
Survey on Fingerprint Spoofing, Detection Techniques and Databases [6]	2015	No	Contact-based
Security and Accuracy of Fingerprint-Based Biometrics: A Review [7]	2019	Few	Contact-based
A Survey on Unknown Presentation Attack Detection for Fingerprint [8]	2021	Few	Contact-based, SWIR, LSCI
Robust anti-spoofing techniques for fingerprint liveness detection: A Survey [9]	2021	Few	Contact-based
FinPAD: State-of-the-art of fingerprint presentation attack detection mechanisms, taxonomy and future perspectives [10]	2021	Yes, <30	Contact-based, SWIR
Fingerprint Liveness Detection Schemes: A Review on Presentation Attack [11]	2022	Yes, <30	Contact-based, SWIR, LSCI, smartphone
**Deep Learning for Fingerprint Presentation Attack Detection: A Survey (Ours)**	2026	Comprehensive (>60)	Contact-based, SWIR, LSCI, FTIR, OCT, smartphone

**Table 3:** Comparison of different PAI generation techniques.

Digital PAI	Artificial Fabrication PAI
Generate high-quality attack instrument	Generate near high-quality attack instrument
High attack potential	Moderate attack potential
Able to attack multiple identities in a single attack	Mostly designed to attack a single identity
Requires more technical knowledge	No need for more technical knowledge
High computation cost	Low computation cost
Low-cost generation	High-cost generation
Very challenging to detect	Easy to detect, particularly with the multi-spectral sensors

**Table 4:** Most utilized and publicly available datasets. We term the different major PAIs shortly: **Silicone—S**, **Gelatin—GE**, **Gelatin—G**, **Latex—L**, **Wood glue—WG**, **Ecoflex—E**, **Liquid Ecoflex—LE**, **Play-Doh—PD**, **Body Double—BD**, **Print—P**, **Replay—R**, **Modasil—M**. **NA** refers to the there is no specific information.

Dataset	No. of Subjects	Bona Fide Samples	Attack Samples	PAI Type
Tsinghua [28]	15	300	470	S
BSL [29]	45	900	400	S, GE, L, WG
LivDet 2009 [33]	254	5500	5500	GE, S and PD
LivDet 2011 [34]	200	3000	3000	GE, E, WG, PD, S and L
LivDet 2013 [35]	225	8000	8000	GE, WG, L, E and M
LivDet 2015 [36]	100	4500	5948	BD, E, P, GE, L, WG and LE
LivDet 2017 [37]	150	8099	9685	GE, WG, L, E, BD and LE
LivDet 2019 [38]	NA	6029	6936	GE, WG, L, E, BD and LE
LivDet 2021 [39]	66	10,700	11,740	GLS20, BD, G, and RFast30
LivDet 2023 [40]	25	5000	3000	NA
ATVS-FFp [41]	17	816	816	S, PD
PBSKD [30]	NA	1000	900	E, GE, L, Crayola, WG, 2D print
ZJUT-EIFD [42]	60	3,551,800	73,500	NA
IIITD [31]	128	4096	8192	P and R
NTNU [43]	200	500	588	P and R
MSU-FPAD [30]	NA	9000	10,500	E and P
COLFISPOOF [32]	NA	NA	7200	P and R
CLARKSON [44]	26	5886	4247	E, P, PD and WG

**Table 5:** Structured comparison of representative deep-learning FPAD paradigms across sensing scenarios and deployment constraints (high-level synthesis).

Paradigm	Capture Type	Typical Backbone/Cue	Strengths	Key Risks	Deployment Notes
End-to-end supervised CNN	Contact/ contactless	Texture- and ridge-detail CNNs (e.g., ResNet variants)	High in-domain accuracy; simple training	Overfitting to sensor/material; leakage risk	Works well for fixed sensors; needs continuous monitoring
Transfer learning	Contact/ smartphone	Pre-trained CNNs + fine-tuning	Data efficiency; faster convergence	Negative transfer under domain shift	Prefer light backbones for on-device inference
Domain generalization/adaptation	Contactless/ cross-sensor	Feature alignment, style/augmentation, meta-learning	Improved cross-domain robustness	Sensitive to protocol; may reduce in-domain accuracy	Best when target sensor unknown or evolving
One-class/ anomaly detection	All (esp. unseen PAI)	Autoencoders, SVDD-style, density models	Better for unseen attacks; security-oriented	Higher bona fide rejection if poorly calibrated	Requires careful thresholding and open-set evaluation
Hybrid (multi-cue/multi-branch)	All	Fusion of texture, frequency, quality, or temporal cues	Robustness via complementary cues	Complexity; harder to interpret	Useful when latency budget allows and attacks are diverse

**Table 7:** Existing contact-based FPAD methods using transfer learning/fine-tuning.

Author	Year	Backbone	Loss Function	Main Contribution
Nogueira et al. [96]	2016	AlexNet, VGG	Binary CE loss	Fine-tuning pre-trained CNNs
Toosi et al. [97]	2017	AlexNet	Binary CE loss	Patch-based voting
Toosi et al. [98]	2017	AlexNet, VGG19	Binary CE loss	Transfer learning on CNN
Ametefe et al. [99]	2021	DenseNet	Binary CE loss	Transfer learning on DenseNet
Rajaram et al. [100]	2024	MobileNet V2	Binary CE loss	Transfer learning on MobileNet V2

**Table 9:** Existing state-of-the-art contactless-based FPAD methods.

Author	Year	Backbone	Loss Function	Type of Image
Hussein et al. [110]	2016	CNN	Binary CE loss	SWIR and LSCI images
Mirzaalian et al. [111]	2019	CNN	Binary CE loss	LSCI images
Kolberg et al. [112]	2020	LSTM network and CNN	Binary CE loss	LSCI images
Spinoulas et al. [113]	2021	CNN	Binary CE loss	Near-infrared (NIR), SWIR, LSCI images
Sun et al. [114]	2023	DenseNet	Dice loss and Binary CE loss	OCT images
Zhang et al. [115]	2024	CNN	Binary CE loss	OCT images

**Table 10:** Existing state-of-the-art anomaly detection-based FPAD methods.

Author	Year	Backbone	Loss Function	Main Contribution
Engelsma and Jain [117]	2019	GAN	Adversarial loss	Trained three GANs on different images
Rohrer and Kolberg [118]	2021	Wasserstein GAN and AutoEncoder	Reconstruction loss	Pre-trained WGAN
Kolberg et al. [120]	2021	AutoEncoder	Reconstruction loss	Trained three AutoEncoders
Liu et al. [121]	2021	AutoEncoder	Reconstruction loss	AutoEncoder based on OCT images
Liu et al. [122]	2023	AutoEncoder	Reconstruction loss	Denoising autoencoder

**Table 13:** Interpretability of fingerprint presentation attack detection methods.

Author	Year	Backbone	XAI Tools
Liu et al. [107]	2023	Self-designed module	Grad-CAM
Dastagiri et al. [147]	2023	Attention-based module	Feature-level interpretation
Yuan et al. [90]	2024	Siamese attention Res-CNN	Grad-CAM
Fei et al. [149]	2024	Self-designed module	Grad-CAM

**Table 14:** Contact-based FPAD benchmark. Columns: *Dataset* lists the evaluation corpus/protocol; *Metric* is reported exactly as in the source; *Value* is the corresponding scalar (single value or per-year aggregate); *Cost* refers to the overall computation cost based on the estimated inference cost of the backbone (Low/Medium/High). Metrics: **Acc** = overall classification accuracy. **ACE** = average classification error $=\frac{1}{2}\left(\mathrm{APCER}+\mathrm{BPCER}\right)$ (lower is better), where **APCER** is the proportion of attacks misclassified as bona fide and **BPCER** is the proportion of bona fide samples misclassified as attacks. **D-EER** = detection equal error rate. An em dash (—) indicates that a comparable single-number metric was not reported by the authors for the stated protocol. All values are quoted as reported and may reflect different protocols.

Method (Year)	Dataset	Metric	Value	Cost
Wang et al. [67], 2015 (DCNN + patch voting)	LivDet 2011/2013	—	—	High
Menotti et al. [69], 2015 (SpoofNet)	LivDet 2011/2013/2015	—	—	High
Kim et al. [70], 2016 (DBN)	LivDet 2011/2013	—	—	Medium
Park et al. [68], 2016 (random-patch CNN)	LivDet 2011	ACE	3.42%	Medium
Lazimul & Binoy [78], 2017 (enhance+CNN)	Private	—	—	Medium
Jang et al. [79], 2017 (contrast+CNN)	Private	—	—	Medium
Chugh et al. [71], 2017 (Inception-v3, minutiae patches)	LivDet 2011/2013/2015	—	—	High
Chugh et al. [30], 2018: Spoof Buster (MobileNet-v1)	LivDet 2015	Acc	99.03%	Low
Pala [80], 2017 (triplet embedding)	Private	—	—	Medium
Jung & Heo [81], 2018 (liveness-map CNN)	Private	Acc		Medium
Nguyen et al. [74], 2018: fPADnet (SqueezeNet+Gram)	LivDet 2011/2013/2015	ACE	2.61%	Low
Park et al. [77], 2018/2019 (Gram/Tiny-FCN)	LivDet 2011/2013/2015	ACE	1.43%	Low
Yuan et al. [82], 2019 (ISE layer CNN)	LivDet 2011/2013	ACE	6.45%/3.70%	Medium
Zhang et al. [83], 2019: Slim-ResCNN	LivDet 2017	Acc	95.25%	Medium
Zhang et al. [84], 2020: FLDNet	LivDet 2015	ACE	1.76%	Medium
Jian et al. [85], 2020 (GA-DenseNet)	Private	—	—	High
Liu et al. [86], 2021: Channel-wise Feature Denoising	LivDet 2017	ACE	2.53%	Medium
Rai et al. [87], 2023: MoSFPAD (MobileNet+SVC)	LivDet 2011-2019	Acc	97.13%	Low
Grosz et al. [88], 2023: ViT Unified	LivDet 2013/2015	Acc	98.87%	High
Nogueira et al. [96], 2016 (AlexNet/VGG fine-tuning)	LivDet 2015	Acc	95.5%	High
Toosi et al. [97], 2017 (AlexNet; patch-based voting)	LivDet 2011/2013	ACE	4.6%	High
Toosi et al. [98], 2017 (AlexNet, VGG19; transfer learning)	LivDet 2011/2013	ACE	3.3%	High
Ametefe et al. [99], 2021 (DenseNet201 transfer learning)	LivDet 2009–2015	Acc	99.8%	High
Rajaram et al. [100], 2024 (CLNet/MobileNetV2 TL)	LivDet 2015	Acc	98.32%	Low
Pereira et al. [101], 2020 (species-invariant adv. learning)	LivDet 2015	APCER	0.76%	Medium
Chugh & Jain [102], 2020: UMG (style transfer)	LivDet 2017	ACE	95.88%	High
Sandouka et al. [103], 2021: Unified GAN + EfficientNetV2	Private	—	—	High
Sandouka et al. [104], 2021: Transformer + CycleGAN	LivDet 2015	Acc	83.12%	High
Lee et al. [106], 2022: CNN + CycleGAN (style transfer)	Private	—	—	High
Liu et al. [107], 2022: CFD (MobileNetV2 + PA-Adaptation)	LivDet 2017	ACE	2.53%	Low
Anshul et al. [108], 2023: Auxiliary Classifier GAN	Private	—	—	High
Rai et al. [109], 2024: Open Patch Generator (GAN)	LivDet 2015/2017/2019	Acc	94.69%	High

**Table 15:** Smartphone FPAD benchmark. Columns: *Dataset/Protocol* lists the evaluation corpus/protocol; *Metric* is reported exactly as in the source; *Value* is the corresponding scalar; *Cost* refers to the overall computation cost based on the estimated inference cost of the backbone (Low/Medium/High). Metrics: **APCER** is the proportion of attacks misclassified as bona fide and **BPCER** is the proportion of bona fide samples misclassified as attacks. **D-EER** = detection equal error rate. An em dash (—) indicates that a comparable single-number metric was not reported by the authors for the stated protocol. All values are quoted as reported and may reflect different protocols.

Method (Year)	Dataset/Protocol	Metric	Value	Cost
Zhang et al. [124], 2016 (Improved CNN)	Private	—	—	Medium
Fujio et al. [125], 2018 (AlexNet)	IIITD fingerphoto	APCER	0.04%	High
Marasco & Vurity [126], 2021 (AlexNet/ResNet18)	Private	—	—	High
Marasco et al. [127], 2022 (Deep color spaces; score fusion)	IIITD fingerphoto	D-EER	2.12%	Medium
Purnapatra et al. [44], 2023 (DenseNet/NasNet; CLARKSON)	CLARKSON, NTNU, Private	D-EER	27.36%, 34.21%, 38.89%	High
Li & Raghavendra [129], 2023 (8 CNNs; deep features)	CLARKSON	D-EER	8.26%	High
Adami et al. [131], 2023 (ResNet18)	CLARKSON, CoLFiSPOOF	APCER	0.63%	Medium
Priesnitz et al. [138], 2023 (CoLFiPAD benchmark)	CoLFiSPOOF	D-EER	4.14%	Medium
Li & Raghavendra [130], 2024 (background influence)	CLARKSON	D-EER	8.26%	High
Priesnitz et al. [133], 2024 (SpoofBuster)	CLARKSON, NTNU, Private	D-EER	21.71%, 24.50%, 31.54%	Low
Liu et al. [134], 2024 (Wavelet AE; unsupervised)	CLARKSON, NTNU, Private	D-EER	22.45%, 25.67%, 33.78%	Medium
Adami et al. [135], 2024 (AE; unsupervised)	CLARKSON, NTNU, Private	D-EER	20.75%, 23.36%, 32.16%	Medium
Li et al. [136], 2024 (Diffusion; unsupervised)	CLARKSON, NTNU, Private	D-EER	18.80%, 22.41%, 29.28%	High
Vurity et al. [139], 2025 (MobileNet; multi color spaces)	Private	—	—	Low
Adami & Karimian [140], 2025 (Swin-UNet; domain adaptation)	CLARKSON, CoLFiSPOOF, IIITD	APCER	1.3%, 0.08%, 0.21%	High
Li et al. [137], 2025 (LLM)	Private	—	—	High

## Data Availability

There is no data to be published.
